# Antioxidant and Potentially Anti-Inflammatory Activity of Anthocyanin Fractions from Pomace Obtained from Enzymatically Treated Raspberries

**DOI:** 10.3390/antiox8080299

**Published:** 2019-08-10

**Authors:** Urszula Szymanowska, Barbara Baraniak

**Affiliations:** Department of Biochemistry and Food Chemistry, University of Life Sciences, Skromna Str. 8, 20-704 Lublin, Poland

**Keywords:** raspberry pomace, anthocyanins, pectinolytic enzymes, antioxidant activity, anti-inflammatory activity

## Abstract

Raspberry pomace was obtained from raspberries subjected to enzymatic maceration using three commercial pectinolytic preparations (Pectinex Ultra SP-L, Pectinex Yield Mash, and Ultrazym AFP-L). Phenolic compounds were extracted and anthocyanin fractions were isolated using the SPE solid phase extraction technique. In the separated anthocyanin fractions, the content of individual compounds was determined by the HPLC technique and the antioxidant activity was assessed with four complementary methods (DPPH and ABTS radical scavenging activity, chelating Fe(II) power, and ferric reducing power). Potential anti-inflammatory properties were also identified as the ability to inhibit the activity of lipoxygenase and cyclooxygenase 2. For these enzymes, the type of inhibition was determined based on the Lineweaver–Burke plot.

## 1. Introduction

Raspberry fruits are very delicate and their storage period is considerably limited, because they become microbiologically infected in a short time, which leads to a decrease in their quality and health benefits [1]. As a result, only small amounts of raspberries are eaten fresh or frozen. Larger quantities are processed into juices, jams, jellies, and syrups [2]. Raspberry juice is the most popular product obtained from these fruits, both on an industrial scale and for domestic use. Enzymes such as pectinases, cellulases, and hemicellulases are commonly used in the production of fruit juices to facilitate extraction of juice via enzymatic degradation of cell wall components. During this process, considerable amounts of raspberry pomace are formed, which consist of pulp, peels, and seeds. The processing or utilization of by-products from the production of food of plant origin, including raspberry pomace, is a serious problem for the food industry [3]. Due to the high consumption and industrial processing of the fruit, the pomaces are generated in large quantities in the food industry. It may cause environmental pollution problems, and as a result, there is growing interest to find alternative uses for the by-products generated by agriculture and the food industry. Furthermore, food wastes or by-products are inexpensive and an easily available source of many bioactive and functional compounds with proven health-promoting properties [4].

By-products from food of plant origin are cheap sources of compounds with antioxidant properties. The extraction of these compounds from such plant wastes is very important for the development of functional food products [5]. Due to the growing interest of consumers in food with high health potential, pomace from berries (including raspberries) may be a good source of bioactive compounds [4]. Raspberry pomace is especially rich in various groups of phenolic compounds, mainlyellagitannins, proanthocyanidins, anthocyanins, flavonols, and smaller amounts of phenolic acids. In particular, anthocyanins and ellagic acid are responsible for health-related properties [6]. It has been reported that raspberry phenolic ingredients have a beneficial effect on human health. They exhibit antioxidant and anti-bacterial activity as well as physiological properties, i.e., anti-allergic, anti-atherosclerotic, anti-inflammatory, antithrombotic, cardioprotective and vasodilatory effects. [7].

Polyphenols, especially anthocyanins, are known as powerful antioxidants. The mechanism of action of these compounds is multidirectional: free radical scavenging, chelation of transition metal ions (copper, iron), inhibition of enzymes involved in the formation of reactive oxygen species (ROS), induction of endogenous antioxidant enzymes, and prevention of lipid peroxidation [8]. The anti-inflammatory activity of natural phenolic compounds (including anthocyanins) may involve several mechanisms: antioxidant effects and neutralization of free radicals, regulation of the activity of inflammation-associated cells, effects on the activity of enzymes involved in the metabolism of arachidonic acid (phospholipase A2, lipoxygenase, cyclooxygenase) and inducible nitric oxide synthase (iNOS), regulation of the production of proinflammatory compounds (e.g., transcription factors, NF-κB, proinflammatory cytokines), and modulation of the expression of proinflammatory genes [9].

Phenolic compounds recovered from pomace can be used as food additives at the stage of food production or as ready-made supplements consumed/taken during meals. This is desirable by consumers, as a large amount of processed finished products is poor in compounds with pro-health properties. As a result, phenolic compounds obtained from pomace may constitute a dietary supplement [10].

The aim of our study was to determine the antioxidant potential and the anti-inflammatory activity expressed as the ability to inhibit the lipoxygenase and cycloxygenase-2 activity of an anthocyanin fraction, isolated from raspberry pomaces, obtained after pectinolytic enzyme-assisted juice pressing. Additionally, the type of inhibition of these enzymes by an anthocyanin fraction from raspberry pomace was determined.

## 2. Materials and Methods

### 2.1. Material

Raspberry fruits (*Rubus idaeus* L. *var. Polana*) were obtained from an organic farm—“Colours of Health”, Tarnogród, Poland.

### 2.2. Sample Preparation

The raspberries (1 kg) were blended for 1 min in a domestic blender (Braun GmbH, Kronberg im Taunus, Germany) and heated in a shaking incubator (INCU-Shaker MINI) to reach the temperature 45 °C. It was constantly shaken and the temperature was sustained with a laboratory thermometer. Enzymes were kindly supplied by Novozymes (Warsaw, Poland) and were used at the average dosage recommended by the manufacturers (10 mL/100 kg mash). Each enzyme was diluted with distilled water to a constant volume of 10 mL before adding to the mashed raspberries [11]. The enzymatic maceration process was run with shaking for 1 h. The raspberry mash was then heated to 85 °C (2 min) to inactivate the enzymes added. After the treatment, the raspberry mashes were cooled, squeezed (Omega squeezer Eujuicers.com 8004, Prague, Czech Republic), and centrifuged (15 min, 4 °C, 9000× *g*). The pomaces were separated, weighed, and stored at –20 °C for further analyses.

We used the following scheme designations:

- C—control (distilled water instead of the enzymes)
- E1—Pectinex Ultra SP-L (polygalacturonase activity)
- E2—Pectinex Yield Mash (pectinmethylesterase activity)
- E3—Ultrazym AFP-L (polygalacturonase and cellulase activity)

### 2.3. Isolation of Phenolic Compounds

The extraction of phenolic compounds was performed according to the method described by Rodriguez-Saona and Wrolstad [12]. A total of 15g of raspberry pomaces were extracted for 1 h, three times, with 30 mL of 90% acidified methanol (0.1% *v/v* HCl). After each cycle, the extracts were centrifuged for 15 min at 9000× *g* at 4 °C and filtered. Acidified methanol was added to the combined supernatants to a volume of 100mL; this was the crude extract. The extraction procedure was followed by methanol removal using a rotary evaporator (RVO 200A, Ingos, Prague, Czech Republic), (40 °C, 700 mPa) and dissolution in 5 mL of deionized acidified water (0.1% *v/v* aqueous solution of HCl). Next, the obtained samples were fractionated using a procedure described by Rodriguez-Saona and Wrolstad [12]. The extracts were passed through Supelco C-18 cartridges (Sigma-Aldrich, Poznań, Poland) activated with acidified methanol followed by 0.1% HCl (*v/v*) in deionized water. Anthocyanins were adsorbed onto the column while carbohydrates, acids, and other water-soluble compounds were removed by flushing with 0.1% HCl. The cartridges were washed with ethyl acetate to remove phenolic compounds other than anthocyanins. Next, anthocyanins were recovered with methanol containing 0.1% HCl (*v/v*). The methanol fractions were evaporated using a rotary evaporator at 40 °C and the solids were dissolved in methanol and used for further analysis as a purified anthocyanin fraction (a).

### 2.4. Analytical Methods

#### 2.4.1. HPLC Analysis of Anthocyanins

Purified anthocyanin fractions were used for the quantitative analysis of anthocyanins via HPLC/DAD as described previously [13].

#### 2.4.2. Antioxidant Activity

Free radical scavenging activity was measured using DPPH and ABTS^•+^ as a source of free radicals according to the methods used by Brand-Williams et al. [14] and Re et al. [15], respectively. The antioxidant activity was expressed as IC50 (the extract concentration required to inhibit 50% of the DPPH or ABTS in the assay medium).

Chelating power was determined using the method described by Guo et al. [16]. It was expressed as EDTA equivalent in mg EDTA per g of fresh weight (FW). The standard curve was prepared in a concentration range of 0–150 μg·mL^−1^ of EDTA (*r*^2^ = 0.996).

Reducing power (FRAP) was determined using the method described by Pulido et al. [17]. It was expressed as a Trolox equivalent (TE) in μmol of Trolox per gram of fresh weight (FW).

#### 2.4.3. Determination of Potential Anti-Inflammatory Properties

The lipoxygenase (LOX) inhibitor activity was measured spectrophotometrically according to the method described by Axelroad et al. [18].

Inhibition of lipoxygenase activity by anthocyanins.

The absorbance was measured after 3 min of incubation, a total of 10 μL of the enzyme solution with different volumes of anthocyanin fractions (10, 25 and 50 µL) and such an amount of phosphate buffer at pH = 7.0, so that the total volume of the mixture was 1.5 mL. The reaction was started by adding 40 μL of the substrate (linoleic acid–LA) solution. The zero sample contained: a phosphate buffer at pH = 7.0 and an appropriate amount of extract (inhibitor) to eliminate the effect of color and the substrate solution. It has been demonstrated that the ability to inhibit LOX activity shows a linear relationship with the amount of extract used in the assay. This dependence allowed for determining the IC 50 value, which determines the concentration of the extract, in mg of FW/mL, needed to inhibit the enzyme activity in 50%.

Cyclooxygenase-2 inhibitor activity was determined spectrophotometrically at 610 nm by measuring the activity of the COX peroxidase subunit using NNN’N’-tetramethyl-p-phenylenediamine (TMPDA) as an electron donor, with own modification as described previously [13].

Inhibition of cyclooxygenase activity by the anthocyanins.

The absorbance changes were measured after 3 min of incubation of the enzyme with different volumes of anthocyanin fractions (10, 20 and 40 μL) and with amount of Tris-maleate buffer, pH = 6.5, so that the total volume of the mixture was 1mL. The zero sample contained: Tris-maleate at pH = 6.5 and the appropriate amount of extract (inhibitor) to eliminate the effect of color. It has been shown that the ability to inhibit COX activity shows a linear relationship with the amount of extract used in the assay. This dependence allowed for determining the IC50 value, which determines the concentration of the extract, in mg of FW/mL, needed to inhibit the enzyme activity in 50%.

#### 2.4.4. Determination of the Type of LOX and COX-2 Inhibition

In order to determine the Michaelis–Menten constant (Km), Vmax and the type of LOX activity inhibition, reactions were carried out using three different substrate concentrations (LA)—1.25; 2.5 and 5.0 mM at constant enzyme concentration (250 U) and the reaction using three different concentrations of LA 1.25; 2.5 and 5.0 mM at a constant enzyme concentration of 250 U and a constant amount of inhibitor (anthocyanins fraction).

In order to determine the Km, Vmax and the type of COX inhibition, reactions were carried out using three different substrate concentrations (arachidonic acid—AA) of 1.25; 2.5 and 5.0 mM and proportional concentrations of TMPD at constant enzyme concentration (0.02 mg/mL) and the reaction using three different concentrations of AA (1.25; 2.5 and 5.0 mM) and proportional concentrations of TMPD at a constant enzyme concentration (0.02 mg/mL), as well as a constant amount of inhibitor (anthocyanins fraction). All determinations were made in triplicate. After the reactions, the double-reciprocal Lineweaver–Burk plot of 1/V versus 1/S was plotted to determine the inhibition type of LOX and COX-2 by anthocyanin fractions from raspberry waste.

### 2.5. Statistical Analysis

All experimental results were means, and the experiments were performed in triplicate (three extracts and three measurements for each extract). The data in the tables and figures represent mean values of standard deviation (*n* = 9). The results were evaluated for statistical significance using univariate analysis of variance (ANOVA) with Statistica 13.0 software (StatSoft, Inc., Tulsa, OK, USA) and Tukey’s post hoc test. Differences were considered significant at *p* = 0.05. In order to show the relationship between anthocyanins concentration and the studied properties, the results were analyzed by Pearson’s linear correlation using Microsoft Excel 2010. Correlation force for |*r*|: <0.2—no linear relationship; 0.2–0.4—weak dependence; 0.4–0.7—moderate dependence; 0.7–0.9—quite strong dependence; >0.9—very strong dependence

## 3. Results

### 3.1. Anthocyanin Composition

Table 1 shows the content of anthocyanins in the purified anthocyanin fraction from the pomace obtained from the control raspberry pulp and those treated with pectinolytic preparations. The concentration of anthocyanin was the lowest in the anthocyanin fractions from the pomaces obtained from the pulp treated with Pectinex Ultra SP-L—E1P(a) and Pectinex Yield-Mash—E2P(a), i.e., 0.25 and 0.24 mg/g FW, respectively, while the highest value was noted for the purified anthocyanin fraction from the control pomace—CP(a)—0.36 mg/g FW.

Three anthocyanin compounds were identified: cyanidin-3-O-sophoroside, which accounts for approximately 67% of the total anthocyanin content, cyanidin-3-O-glucoside, and cyanidin-3-O-rutinoside. Cyanidin-3-O-glucoside accounted for 24% of the total anthocyanin content in the purified anthocyanin fraction from the control pomace—CP(a) and 30.5% of the total anthocyanin content in the anthocyanin fraction from the pomace obtained from raspberries treated with Ultrazym AFP-L-E3P(a). The content of cyanidin-3-O-rutinoside was the highest (18 μg/g FW) in the purified anthocyanin fraction from CP(a) and the lowest (3.75 μg/g FW) in the purified anthocyanin fraction from the pomaces obtained from pulp treated with Pectinex Yield Mash E2P(a).

### 3.2. Antioxidant Properties of Purified Anthocyanin Fractions Isolated from Raspberry Pomace

**Figure 1:**
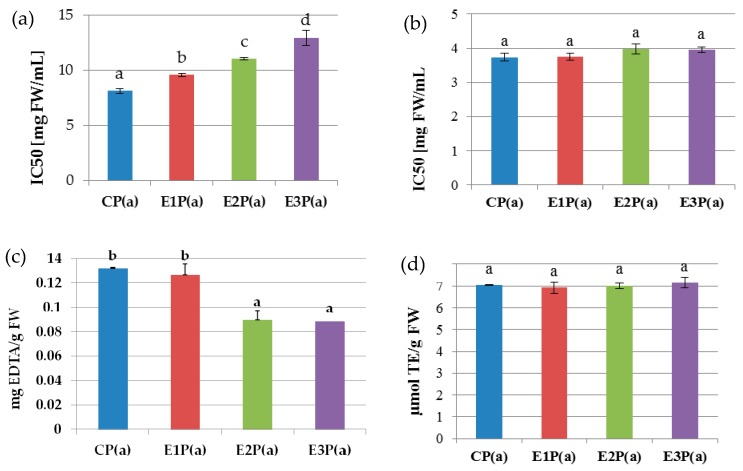
Antioxidant properties of purified anthocyanin fractions from pomace made from raspberries subjected to enzymatic maceration: (**a**) ability to neutralize DPPH^•^ radicals (**b**) ability to neutralize the radicals of ABTS^•+^, (**c**) ability to chelate iron ions, (**d**) reduction power (FRAP). Mean values marked with the same letters do not differ significantly in terms of *p* ≤ 0.05. Symbols: CP(a)—purified anthocyanin fraction from pomace obtained from control raspberry pulp; E1P(a)—purified anthocyanin fraction from pomace obtained from raspberry pulp macerated with Pectinex Ultra SP-L; E2P(a)—purified anthocyanin fraction from pomace obtained from raspberry pulp macerated with Pectinex Yield-Mash; E3P(a)—purified anthocyanin fraction from pomace obtained from raspberry pulp macerated with Ultrazym AFP-L.

Figure 1a shows the antioxidant properties of the anthocyanin fraction isolated from the pomaces. The highest ability to neutralize DPPH radicals was exhibited by anthocyanins isolated from the control pomace—CP(a) (IC50 = 8.15 mg FW/mL), whereas the lowest value was determined for anthocyanins isolated from the pomaces obtained from raspberry pulp treated with Ultrazym AFP-L – E3P(a) – IC50 = 12.92 mg FW/mL). The statistical analysis showed a high negative correlation between the IC50 value and the anthocyanin concentration (*r* = −0.89).

The antiradical properties of the anthocyanin fraction from the pomaces determined against ABTS (Figure 1b) were similar for all analyzed samples. The mean value of the IC50 coefficient was 3.85 mg FW/Ml and was significantly negatively correlated with the content of anthocyanins (*r* = −0.96).

The ability to chelate Fe(II) by the purified anthocyanin fractions isolated from the pomaces is shown in Figure 1c. This activity for the control pomace PC(a) and that obtained from the raspberry pulp treated with Pectinex Ultra-SPL-E1P(a) was 0.13 and 0.126 mg EDTA/g FW, respectively. It was statistically significantly higher than in the case of pomaces obtained from the raspberry pulp treated with Pectinex Yield Mash—E2P(a) and Ultrazyme AFP-L-E3P(a) (0.089 and 0.088 mg EDTA/g FW). There was a high positive correlation between the content of anthocyanins in the analyzed samples and the ability to chelate iron(II) ions (*r* = 0.97).

The reduction power (FRAP) of the anthocyanin fractions from the pomaces (Figure 1d) did not differ significantly between all analyzed samples and was on average 7.0 μM TE/g FW. The values of the determined reduction power were weakly negatively correlated with the anthocyanin concentration (*r* = −0.37).

### 3.3. Potential Anti-Inflammatory Properties of Purified Anthocyanin Fractions Isolated from Pomaces Obtained from Raspberries Subjected to Enzymatic Maceration

**Figure 2:**
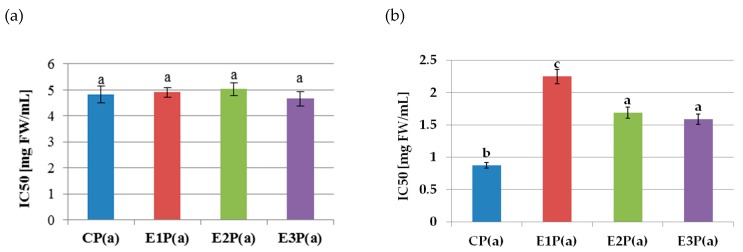
Inhibition of the activity of (**a**) lipoxygenase, (**b**) cyclooxygenase-2, by purified anthocyanin fractions from pomace obtained from raspberries subjected to enzymatic maceration. Mean values marked with the same letters do not differ significantly in terms of *p* ≤ 0.05. Symbols: CP(a)—purified anthocyanin fraction from pomace obtained from control raspberry pulp; E1P(a)—purified anthocyanin fraction from pomace obtained from raspberry pulp macerated with Pectinex Ultra SP-L; E2P(a)—purified anthocyanin fraction from pomace obtained from raspberry pulp macerated with Pectinex Yield-Mash; E3P(a)—purified anthocyanin fraction from pomace obtained from raspberry pulp macerated with Ultrazym AFP-L.

The lipoxygenase inhibitory activity of the purified anthocyanin fractions isolated from the pomaces is shown in Figure 2a. The values of the IC50 coefficients did not differ significantly and amounted to 4.85 mg FW/mL on average. On the basis of Pearson’s correlation analysis, there was no relationship between the ability to inhibit LOX activity (IC50 value) and the content of anthocyanins (*r* = −0.041). The anthocyanin fractions from the pomace showed strong COX-2 inhibitory properties (Figure 2b). The lowest IC50 value (and thus the highest inhibitory activity) of 0.87mg FW/mL was determined for the purified anthocyanin fraction from the control pomace—PC(a). In turn, the highest (the lowest inhibitory activity) value (2.25 mg FW/mL) was found for the anthocyanin fraction from the pomace obtained from the raspberry pulp treated with the Pectinex Ultra SP-L—E1P(a). A weak negative correlation between anthocyanins content and the ability to inhibit COX-2 activity (IC 50 value) was also demonstrated (*r* = −0.38).

**Figure 3:**
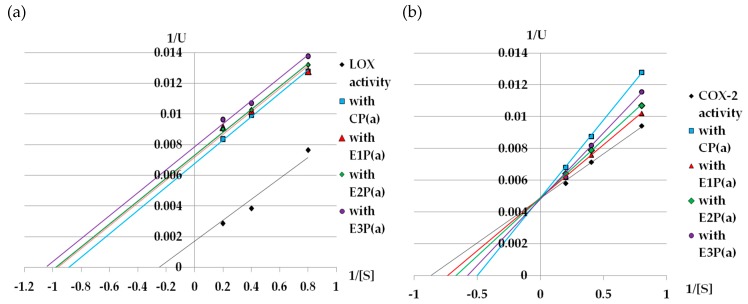
Lineweaver–Burk plot of (**a**) LOX, (**b**) COX-2 inhibition by purified anthocyanin fractions from pomace obtained from raspberries subjected to enzymatic maceration. Symbols: CP(a)—purified anthocyanin fraction from pomace obtained from control raspberry pulp; E1P(a)—purified anthocyanin fraction from pomace obtained from raspberry pulp macerated with Pectinex Ultra SP-L; E2P(a)—purified anthocyanin fraction from pomace obtained from raspberry pulp macerated with Pectinex Yield-Mash; E3P(a)—purified anthocyanin fraction from pomace obtained from raspberry pulp macerated with Ultrazym AFP-L.

Lineweaver–Burk graphs were prepared to determine the kinetic parameters (Km and Vmax) of lipoxygenase treated with purified anthocyanin fractions from pomace. Based on these graphs (Figure 3a), it can be concluded that the purified anthocyanin fractions from the pomace inhibit LOX in an uncompetitive way (Table 2a).

Figure 3b shows the Lineweaver–Burk plot for the inhibition of COX-2 activity by the purified anthocyanin fractions from the raspberry pomace. The Michealis–Menten constant for the reaction without the inhibitor (control reaction) was 1.14mM, while the value of Vmax for this reaction was 200. All inhibitors caused an increase in K_m_, but they did not change the Vmax value, which suggests that they are competitive inhibitors (Table 2b).

## 4. Discussion

In this study, three commercial enzymatic preparations: Pectinex Ultra SP-L, Pectinex Yield Mash and Ultrazym AFP-L were used for maceration of raspberry pulp and then juice was pressed. The purified anthocyanins fraction from the pomace being a waste product after juice extraction was analyzed.

Most of the available publications concern either the influence of pectinolytic preparations on the juice yield and its properties (polyphenol content and antioxidant activity), or the analysis of pomace obtained from fruits after industrial juice pressing or the possibility of recovering bioactive compounds from pomaces treated with pectinolytic enzymes [11,19,20,21,22,23,24,25,26,27,28,29].

The juice color is one of the basic quality indicators. It depends on: raw material, crumbling methods, but also enzymatic processing parameters. The substances responsible for color are natural plant dyes such as carotenoids, anthocyanins, betalains, chlorophylls. These natural dyes are found inside plant cells, therefore enzyme preparations causing disintegration of the cell wall (cellulolytic and pectinolytic) allow for the improvement of dye production [22].

To receive juices from colorful fruits (black currants, strawberries, raspberries and cherries), the so-called hot enzymatic process is recommended. The pulp is heated to 45–50 °C, followed by the process of its enzymation [23]. Nevertheless, significant amounts of polyphenols, including anthocyanins, remain in the pomace. This is confirmed by studies by Kopponen et al. in which the total anthocyanin yield for unprocessed bilberries and black currant was 28–51% and 42–66%, respectively. Hence, even up to 70% of berry anthocyanins are still in pomaces [24].

The concentration of phenolic compounds in pomace can be up to twice as high as in raspberry pulp, if they are expressed in the same units, e.g., in mg/g fresh weight. However, it should be borne in mind that an average of 4–5 g of raspberries is processed to obtain 1 g of pomace.

Compared to raspberries that are very delicate, even larger quantities of bioactive compounds may remain in pomace from other berry fruits. According to Holtung et al. [25] the total content of polyphenols and anthocyanins in blackcurrant pomace was about 3 times higher than in fruits and about 4 times higher than in the obtained juice. Fruits with a dark skin, such as blackcurrant, blueberries, elderberry, bird cherry, etc., contain more anthocyanins than raspberry pomace. However, what constitutes a disadvantage of raspberries as fruit, namely their impermanence and delicacy, in the case of pomace is actually an advantage. It is much easier to extract anthocyanins from raspberry than from black currant or blueberry, which have very dark but also thick skin. Isolation of anthocyanin pigments from these fruits is more time-consuming and expensive.

Thus, the efficiency of enzymatic treatment depends on the type of raw material, the type of enzyme preparation and the applied processing conditions of the raw material [19].

The composition of anthocyanins from raspberry pomace is similar to the composition in whole raspberry fruit. In all 15 analyzed raspberry varieties, Chen et al. [30] identified the presence of cyanidin-3-*O*-glucoside and cyanidin-3-*O*-rutinoside. In nine varieties, there was also cyanidin-3-*O*-sophoroside, cyanidin-3-*O*-glucosylruthinoside, and pelargonidin-3-*O*-glucoside; in the others, cyanidin-3-*O*-xylosylorutoside was found instead. The literature data show that, depending on the cultivar and cultivation conditions, raspberry fruit (*Rubus idaeus* L.) may contain different proportions of cyanidin-3,5-di-*O*-glucoside, cyanidin-3-*O*-sophoroside, cyanidin-3-*O*-glucosylrutinoside, cyanidin-3-*O*-glucoside, cyanidin-3-*O*-rutinoside, pelargonidin-3-*O*-sophoroside, pelargonidin-3-*O*-glucoside, pelargonidin-3-*O*-glucosylrutinoside, and pelargonidin-3-*O*-rutinoside [31,32,33]. Cyanidin-3-*O*-sophoroside is known to be typical for European cultivars [34]. Mildner-Szkurlarz et al. [35] used dried raspberry pomace to enrich muffins. The dominant fraction of polyphenols was represented by anthocyanins, with cyanidin-3-*O*-sophoroside predominating in the samples and contributing to nearly 42% of the total amount of polyphenols. The other anthocyanins are cyanidin-3-*O*-glucosyl-rutinoside, cyanidin-3-*O*-glucoside, and cyanidin-3-*O*-rutinoside. These results are in agreement with our study.

When processing berries into juices using pectin and cellulolytic preparations, not only quantitative but also qualitative changes in phenolic compounds, including anthocyanins take place. In our study maceration of raspberry pulp with all enzymatic preparations affected the anthocyanin profile in the obtained pomace. Koponen et al. [24] have used preparations Econase CE, Biopectinase CCM, Pectinex Smash XXL, and Pectinex BE 3-L for the processing of berry pulp. The addition of enzymes caused an increase in the amount of anthocyanidins (aglycons) in the juice obtained. This is a small amount of agglomeration in the control juice. This phenomenon may result from the presence of a small amount of glycosidases in the used enzyme preparations or from the activity of endogenous hydrolases released from cells during juice production, as evidenced by the presence of a small amount of aglycons in the control juice. Landobo and Meyer, (2004) after using 10 different enzymatic preparations, obtained an increase in the recovery of anthocyanins dyes in juices from blackcurrant, while the anthocyanin profile did not change [26]. In turn, in the studies of Buchert et al. (2005) Pectinex Ultra SP-L, Pectinex Smash, Pectinex BE 3-L and Biopectinase CCM, caused the increase in the total content of anthocyanins in the bilberry and blackcurrant juices by 13–41% and 18–29%, respectively, and influenced the anthocyanin profile [11].

Phenolic compounds, and especially anthocyanins, are responsible for the antioxidant activity of fruits and their products, including pomaces. Pomace, which is a waste product in the process of juice pressing, retains significant amounts of phenolic compounds. Although it is not intended for direct consumption, it may be a rich source of bioactive compounds with potential health-promoting properties. The results of the present study indicate high antioxidant potential of the anthocyanin fraction from the raspberry pomace. The antioxidant activity of phenolic compounds is related to their ability to scavenge free radicals, donate hydrogen atoms or electron, or chelate metal cations. The anthocyanin and anthocyanidin health properties are associated with their peculiar chemical structure, as they are very reactive towards ROS due to their electron deficiency. The antioxidative properties of anthocyanidins have been recently explored; most of the widely distributed anthocyanidins and anthocyanins show higher scavenging activity than that of the well-known strong antioxidants trolox and catechol [36].

In our own research, in terms of antioxidant properties of the anthocyanin fraction, Ultrazym AFP-L proved to be the most effective preparation. After its application, the ability to neutralize the DPPH radical and the ability to chelate iron(II) ions were the lowest, and hence, the conclusion that the most anthocyanins, i.e., strong antioxidants, were released into the juice.

Puupponen-Pimiä et al. (2008) reported that out of the 9 enzyme preparations used to berry pulp maceration, after applying Pectinex Smash XXL, they determined the highest anthocyanin content and the highest antioxidant capacity, and the lowest after using Pectinex BE 3-L. Thus, for each raw material, an enzymatic preparation should be selected individually [28].

As demonstrated by DPPH assay results, among pomaces obtained from berry fruits, blackberry exhibits the highest antioxidant activity. Četojević-Simin et al. [37] reported that a pomace extract from the blackberry cultivar *Thornfree* demonstrated stronger DPPH radical scavenging activity (2.12 mmol TEAC g ^−1^) than a *Čačanska bestrna* pomace extract (1.03 mmoL TEAC g ^−1^). Similarly, Jazić et al. [38] found that the extract of a wild variety of blackberry effectively inhibited free radicals, i.e., IC50 = 127.76 µg/ mL in the DPPH assay and IC50 = 26.53 µg/mL in the ABTS assay. In turn, the antiradical activity of a raspberry pomace extract evaluated by the ABTS assay was 1.17 mM Trolox equivalents at the concentration of 3.33 mg/mL [39]. Četojević-Simin et al. [37] investigated raspberry pomaces and found that a Willamette pomace extract (EC50 = 0.042 mg/mL) demonstrated stronger DPPH radical-scavenging activity than a Meeker pomace extract (EC50 = 0.072 mg/mL). Comparing our results with those obtained by other researchers, it is necessary to take into account the fact that in our work we analyzed the purified anthocyanin fraction rather than the entire polyphenol pool present in the pomace. Total antioxidant activity may be a result of synergic interactions among the antioxidant compounds [36].

The results obtained by Vulić et al. [40] indicate that the reducing power of berry pomace extracts increased with increasing concentrations. A strawberry pomace extract and a blackberry pomace extract showed the highest (RP_0.5_ = 0.35 mg/mL) and the lowest (RP_0.5_ = 0.57 mg/mL) reducing power, respectively, while raspberry pomace exhibited an intermediate value.

In addition to the enzymatic maceration of the pulp, the process of enzymatic processing of the pomace can also be used. The advantage of this operation is to obtain two different products in one process, allowing for greater flexibility in their development. Laroze et al. (2010) used 12 enzyme preparations for the maceration of raspberry pomace. Of these, three (Grindamyl CA 150, Maxoliva and Rohapect Max) increased the amount of polyphenols in the extract, five contributed to a significant reduction in the content of phenolic compounds, while the remaining ones did not cause statistically significant changes. The lack of efficacy of some enzyme preparations in increasing the recovery of polyphenols can be explained by the high content (63%) of lignin in raspberry pomace [27].

The results of our studies indicate that anthocyanins isolated from pomace obtained after enzymatic maceration of raspberry pulp may be potential inhibitors of LOX and COX-2, i.e., enzymes involved in inflammation induction. An interesting direction in the latest anti-inflammatory studies seems to be the search for compounds that can simultaneously inhibit cyclooxygenase-2 (COX-2) and 5-lipoxygenase (5-LOX), which are the so-called COX/5-LOX double inhibitors. Many recent research results suggest that phenolic compounds, in particular flavonoids, may be simultaneous inhibitors of lipoxygenase and cyclooxygenase [9,41,42]. Due to the similarity of the structure of animal lipoxygenases and lipoxygenase from soybeans, the latter is very often used in research seeking natural 5-LOX inhibitors [43].

Anthocyanins are also mentioned as compounds exhibiting anti-inflammatory activity. Many processed fruit products (chokeberries, blueberries, raspberries) have traditionally been used as an auxiliary in the treatment of colds and flu-like conditions. Recent scientific studies confirm that anthocyanins have the ability to inhibit the activity of arachidonic acid-metabolizing enzymes. It has been shown that anthocyanins from saskatoon berries [44], cherries, blueberries, cranberries, elderberries, and raspberries [45] are effective COX-2 inhibitors. Natural color pigments, e.g., anthocyanins from blueberries, cranberry juice, chokeberry concentrate [46], and pomegranate [36] often inhibit the activity of lipoxygenase, effectively preventing the formation of leukotrienes. However, in vitro tests do not take into account factors that occur in vivo, such as the intracellular location of COX-1 and COX-2 and the potential differences in local intracellular concentrations of the analyte, laboratory results (in vitro) do not reflect the clinical situation.

Kinetic studies conducted in the presence of different substrate concentrations reveal an uncompetitive and competitive mode of LOX and COX-2 inhibition, respectively, in the presence of the purified anthocyanin fraction from pomaces obtained after pectinolytic enzyme-assisted juice pressing (Figure 3a,b). These results are confirmed by our previous studies [13]. Knaup et al. [46] showed that various glycosides of delphinidin, cyanidin, peonidin, and malvidin also act as uncompetitive inhibitors of lipoxygenase, which confirms the results obtained in this work.

## 5. Conclusions

The use of pectinolytic enzymes during juice extraction significantly reduces the amount of bioactive compounds remaining in the pomace. Nevertheless, raspberry pomace is still a rich source of anthocyanins with strong in vitro antioxidant and anti-inflammatory effects. Raspberry pomace can be an effective ion binder since it is a rich source of polyphenols. Berry fruit pomace contains abundant phenolic compounds, which indicates that this by-product can be useful as a raw material for production of new value-added food, pharmaceuticals, and cosmetic formulations. The results obtained in this study show that anthocyanins from raspberry pomace are a natural source of effective and safe bioactive compounds for development of functional foods for prevention and treatment of free radical-related and inflammatory diseases. It is obvious that in vitro studies require further in vivo verification, first on animal models, then with volunteers. In vitro studies are model studies of cognitive nature and do not reflect the complexity of the relationships and interactions that occur in vivo. Therefore, the next step should be to determine the potential bioavailability of anthocyanins after in vitro digestion and to study their antioxidant and anti-inflammatory properties. Further research is also needed to determine the mechanisms of action of extracts, individual compounds, interactions between them and their potential application on a wider scale.

## Figures and Tables

**Table 1:** Content of individual anthocyanins in the purified anthocyanin fractions isolated from pomace obtained from raspberries subjected to enzymatic treatment by HPLC.

Compound	Content of Individual Anthocyanins [µg/g FW] *			
	CP(a)	E1P(a)	E2P(a)	E3P(a)
cyanidin-3-*O*-sophoroside	255.6 ± 14.9 ^c^	178.5 ± 12.85 ^a^	156 ± 11.05 ^a^	214.4 ± 14.74 ^b^
cyanidin-3-*O*-glucoside	86.4 ± 5.04 ^b^	66.25 ± 4.77 ^a^	72 ± 5.1 ^a^	97.6 ± 6.71 ^b^
cyanidin-3-*O*-rutinoside	18.0 ± 1.05 ^c^	3.75 ± 0.27 ^b^	12 ± 0.85 ^a^	12.8 ± 0.88 ^a^
Sum [mg/g FW] ^x^	0.36 ± 0.021 ^b^	0.25 ± 0.018 ^a^	0.24 ± 0.017 ^a^	0.32 ± 0.022 ^b^

^x^ calculated as cyanidin-3-O-glucoside; * Average in lines marked with the same letters do not differ significantly in terms of *p* ≤ 0.05; Symbols: CP(a)—purified anthocyanin fraction from pomace obtained from control raspberry pulp; E1P(a)—purified anthocyanin fraction from pomace obtained from raspberry pulp macerated with Pectinex Ultra SP-L; E2P(a)—purified anthocyanin fraction from pomace obtained from raspberry pulp macerated with Pectinex Yield-Mash; E3P(a)—purified anthocyanin fraction from pomace obtained from raspberry pulp macerated with Ultrazym AFP-L.

**Table 2:** Km, Vmax and inhibition type of (a) LOX and (b) COX-2 activity.

**(a)**			
Inhibitor	Km (mM)	Vmax	LOX inhibition type
without inhibitor	4.0	588.24	-
CP(a)	1.136	148.15	uncompetitive
E1P(a)	1.064	138.89	uncompetitive
E2P(a)	1.03	126.58	uncompetitive
E3P(a)	1.05	137.93	uncompetitive
**(b)**			
Inhibitor	Km (mM)	Vmax	COX-2 inhibition type
without inhibitor	1.14	200	-
CP(a)	2.0	200	competitive
E1P(a)	1.37	200	competitive
E2P(a)	1.515	200	competitive
E3P(a)	1.724	200	competitive

Symbols: CP(a)—purified anthocyanin fraction from pomace obtained from raspberry control pulp. E1P(a)—purified anthocyanin fraction from pomace obtained from raspberry pulp macerated with Pectinex Ultra SP-L; E2P(a)—purified anthocyanin fraction from pomace obtained from raspberry pulp macerated with Pectinex Yield-Mash; E3P(a)—purified anthocyanin fraction from pomace obtained from raspberry pulp macerated with Ultrazym AFP-L.
